# Knowledge of breast cancer among patients undergoing breast cancer treatment at a Tertiary Hospital in Ghana

**DOI:** 10.3332/ecancer.2024.1808

**Published:** 2024-12-04

**Authors:** Josephine Nsaful, Florence Dedey, Kirstyn E Brownson, Ruth Y Laryea, Nathaniel Coleman, John Tetteh, Mohammed Albezel Sheriff, Joe-Nat Clegg-Lamptey, Benedict N L Calys-Tagoe

**Affiliations:** 1 Department of Surgery, University of Ghana Medical School, Korle Bu, PO Box 4236, Accra, Ghana; 2 Department of Surgery, Korle Bu Teaching Hospital, PO Box 77, Accra, Ghana; 3 Huntsman Cancer Institute, Salt Lake City, UT 84112, USA; 4 Department of Surgery, University of Utah School of Medicine, Salt Lake City, UT 84132, USA; 5 Department of Medicine and Therapeutics, University of Ghana Medical School, Korle Bu, PO Box 4236, Accra, Ghana; 6 Department of Obstetrics and Gynaecology, University of Ghana Medical School, Korle Bu, PO Box 4236, Accra, Ghana; 7 Department of Community Health, University of Ghana Medical School, Korle Bu, PO Box 4236, Accra, Ghana; a https://orcid.org/0000-0001-6808-9256

**Keywords:** breast cancer, knowledge, risk factors, treatment options, symptoms

## Abstract

Breast cancer is the leading cause of cancer in females worldwide. Western Africa has one of the highest mortality rates globally partly due to late presentation, often attributed to deficits in patient knowledge about the disease. A cross-sectional study was conducted at the Korle Bu Teaching Hospital among breast cancer patients. A structured questionnaire was utilised to collect data on patient demographics; sources of information on breast cancer; risk factors; symptoms; and treatment options. A chi-square test assessed the relationship between the participants’ levels of knowledge and demographic characteristics. Univariate and multivariate analyses determined which sociodemographic factors predicted knowledge. p values <0.05 were considered statistically significant. This study enrolled 636 participants with a mean age of 52.6 ± 12.1 years. Television (TV) (63.4%) and radio (44.6%) were the main sources of information about breast cancer. Thirty-two percent of participants knew that family history was an associated risk factor for developing breast cancer. Eighty-three percent of the patients were familiar with self-breast examination, but only 42% of them practiced it. While 76% of participants knew that a breast mass could represent breast cancer, only 13%, 12% and 6% of participants identified nipple discharge, breast skin changes and changes in breast size as concerning symptoms of breast cancer, respectively. Only 7% of patients were aware of breast conservation as a treatment option. A higher educational level and higher monthly income were identified as predictors of a better level of knowledge, while age older than 60 years and advanced-stage disease correlated with decreased knowledge about breast cancer. Breast cancer awareness campaigns in Ghana should be expanded to include all Ghanaians, specifically women of lower socioeconomic status and older than 60 years. Educational messages should emphasise symptoms other than breast masses and the feasibility of breast-conserving surgery as a treatment option for early-stage disease.

## Background

Breast cancer is the leading cause of cancer in females worldwide [1]. Western Africa has one of the highest breast cancer mortality rates worldwide [2]. The incidence of breast cancer is projected to increase by 31% of the current estimated 2.3 million cases worldwide by 2040, with less developed countries carrying the highest mortality burden [2]. This high mortality rate is partly due to late presentation, which has been attributed to a lack of knowledge regarding breast cancer [3, 4].

A systematic review of the scientific literature on breast cancer knowledge in Sub-Saharan Africa revealed a dearth of publications on this topic, with the available publications suggesting poor knowledge and infrequent practice of breast self-examination among women in the subregion [5]. Knowledge about symptoms of breast cancer and breast cancer risk factors has also been found to be poor among individuals residing in low- and middle-income countries (LMICs) [6]. There are a few publications from Ghana depicting poor knowledge about breast cancer among the general public [7, 8] and a few qualitative studies exploring the viewpoint of breast cancer patients [9, 10]. Therefore, there is a need to further explore the knowledge base of Ghanaian women diagnosed with breast cancer.

In recent years, a surge in breast cancer awareness campaigns has occurred, particularly during the month of October. Via traditional and social media avenues, women are encouraged to become involved in breast screening. Monthly breast self-examination is specifically recommended. Education about breast cancer symptoms and risk factors for developing breast cancer is also advertised via traditional and social media outlets during this month. Breast cancer walks are organised in various parts of the country, talks and lectures are delivered to various social groups and walk-in clinics offering clinical breast examinations are established during the month. The impact of these activities needs to be investigated. With this in mind, this study endeavored to quantitatively assess sources of information on breast cancer incidence, level of knowledge regarding breast cancer symptoms, risk factors and treatment options among patients receiving breast cancer treatment at a tertiary hospital in Ghana.

## Methods

This was a cross-sectional study conducted at the Korle Bu Teaching Hospital (KBTH) among breast cancer patients as part of a project that sought to determine the level of satisfaction of breast cancer patients with their cancer care [11]. KBTH is a tertiary hospital in Ghana that provides multidisciplinary breast cancer care and is a major referral centre for the country. All participants were females with a histologically confirmed breast cancer diagnosis who had been receiving breast cancer treatment at KBTH. Male patients were excluded. The data were collected from January to December of 2022. All consecutive breast cancer patients at the KBTH surgical Outpatient Department (OPD), chemotherapy suite and radiotherapy OPD were approached, and those who met the inclusion criteria were recruited after providing written informed consent. A structured, interviewer-administered questionnaire was utilised to collect the data. The questionnaire covered patient demographics as well as sources of information on breast cancer and knowledge of breast cancer risk factors, symptoms and treatment options. The survey also assessed participant awareness of breast self-examination.

### Statistical analysis

Stata^®^ 17 software was used to analyse the data. Continuous variables were converted into categorical variables and are presented as frequencies and percentages. Knowledge of breast cancer was based on the total number of questions answered correctly out of ten questions assessing knowledge of breast cancer risk factors, four questions assessing knowledge of breast cancer symptoms and five questions assessing knowledge of breast cancer treatment options (Table 1). Each correct answer was scored as 1, and each incorrect answer was scored as 0. The total scores were converted to percentages. The level of knowledge in each domain was categorised as excellent (≥75% of questions answered correctly), satisfactory (50%–74% of questions answered correctly) or poor (<50% of questions answered correctly). A chi-square test was performed to assess the relationship between the participants’ levels of knowledge and demographic characteristics. Univariate and multivariate analyses were carried out using ordered logistic regression analysis to determine which sociodemographic factors were associated with an excellent, satisfactory or poor level of knowledge regarding risk factors, symptoms and treatment options for breast cancer. All *p* values <0.05 were considered to indicate statistical significance.

## Results

### Patient demographics

The study involved 636 participants, the majority of whom (58%) were 50 years of age or older. The mean age was 52.6 ± 12.1 years. Approximately 83% of the participants had either a junior high school (JHS) or middle school certificate or had completed a higher level of education. Most (68.9%) patients who participated in this study were employed. Over one-third (37.7%) earned less than GHC500 United States Dollar (USD 56 dollars) a month. More than half (56.5%) of the participants were diagnosed with stage 3 breast cancer at the time of initial presentation to KBTH (Table 2).

### Basic knowledge of breast cancer

Ninety-one percent (91.3%) of participants reported that they had heard of breast cancer prior to their diagnosis. Sixty-five percent (64.9%) knew that men could have breast cancer, 7.7% believed that men could not develop breast cancer, and the rest did not know. Seventy-nine percent (78.8%) believed that breast cancer was curable, 13.8% believed that it was not curable and the rest did not know. While 82.7% had heard of breast self-examination, only 42% practiced breast self-examination.

### Sources of information on breast cancer

Patients reported receiving this information about breast cancer from multiple sources. Sixty-three percent (63.4%) of patients learned about breast cancer from TV, 44.6% from the radio, 24.5% from friends, 22.7% from healthcare workers, 22.7% from relatives, 8.5% from print media and 8.4% from the internet.

### Knowledge of breast cancer risk factors, symptoms and treatment options

Table 1 shows that patient knowledge of breast cancer and related risk factors, symptoms and treatment options was generally quite poor. The majority of patients did not recognise family history (60%), personal history (91%), use of hormone replacement therapy (89%), lack of breastfeeding (87%), nulliparous status (89%), early menarche (91%) or late menopause (91%) as risk factors for breast cancer. Only 12% of the participants identified skin changes, 13% identified nipple discharge and 6% identified changes in breast size as concerning symptoms of breast cancer diagnosis. Only 7% of patients knew about breast-conserving surgery as a surgical treatment option, and 11% of patients had knowledge about the importance of endocrine therapy in the treatment of breast cancer.

There was a significant association between knowledge of risk factors and education level and monthly income (*p* value <0.05). Excellent knowledge of breast cancer risk factors was found to be highest among those who had attained tertiary education (2.7%), and this was also highest among breast cancer patients who earned between GHS 2,001 and 5,000 (4.6%) each month (Table 3).

Regarding knowledge of breast cancer symptoms, there were significant associations between knowledge of breast cancer symptoms and patient age, education level, breast cancer stage and monthly income (*p* value <0.05). Specifically, patients ≤39 years of age (20.9%) and those with a tertiary education (23.0%) were more likely to receive an excellent score. Additionally, patients with an income level of 2,001–5,000 GHS/month and patients presenting with clinical stage I or II breast cancer (28% and 11%, respectively) were more likely to receive an excellent score in regard to knowledge of breast cancer symptoms than were the other study participants (Table 4).

Finally, there were significant associations between knowledge of breast cancer treatments and education level, breast cancer stage and patient monthly income (*p* value <0.05). Those with a higher level of education (14.4%), as well as those with a higher monthly income (22.2%), had better knowledge of treatment options. Those who had Stage III & IV disease demonstrated poor knowledge of treatment options, while those with Stage I & II disease had greater proportions of patients with satisfactory to excellent knowledge of treatment options (Table 5).

### Univariate and multivariate analysis of factors associated with knowledge of breast cancer

According to univariate analysis, age and average monthly income were significantly associated with breast cancer patient knowledge about breast cancer risk factors. Individuals aged >60 years were less likely (odds ratio (OR): 0.31; 95% (012–0.83)) to be knowledgeable about breast cancer risk factors than were those aged ≤39 years. Additionally, those who earned between 2,001 and 5,000 GHS per month (225–562 USD) were approximately 2.5 times (OR: 2.49; 95% (1.10–5.62)) more likely to have a score of satisfactory or excellent regarding knowledge of breast cancer risk factors than were those who earned <500 GHS per month. However, following multivariate analysis, the only factor that was significantly associated with knowledge about breast cancer risk factors was the level of education. A person with a tertiary education was approximately six times (OR: 6.27; 95% (1.14–34.39)) more likely to have a satisfactory-to-excellent knowledge score regarding breast cancer risk factors than patients without formal education (Table 6).

According to the univariate analysis, age (*p* = 0.001), highest education level (*p* = 0.001), average monthly income (*p* = 0.001) and cancer stage (*p* = 0.003) were significantly associated with knowledge of breast cancer symptoms. However, after multivariate analysis, only age, educational level and average monthly income remained significant. Specifically, breast cancer patients aged >60 years were less likely (OR = 0.34; 95% (0.16–0.72)) to have satisfactory to excellent knowledge of symptoms of breast cancer than were those younger than 40 years. Additionally, the greater the patient’s level of education is, the greater their knowledge of symptoms of breast cancer. Persons with a tertiary education were approximately 11 times (OR: 10.44; 95% (1.32–82.73)) more likely to have satisfactory to excellent knowledge of the symptoms of breast cancer than were those with no formal education. Finally, individuals who earned more than 2,000 GHS per month were 3–13 times more likely to have a satisfactory to excellent knowledge score on breast cancer symptoms than were breast cancer patients who earned less than 500 GHS each month (Table 7).

Finally, according to the univariate analysis, education level (*p* = 0.004), place of residence (*p* = 0.034), average monthly income (*p* = 0.001) and clinical stage (*p* = 0.006) were significantly associated with knowledge of breast cancer treatment options. However, only the cancer stage was significantly associated with knowledge of breast cancer treatment options according to multivariate ordinal logistic regression. Patients with stages III & IV disease were less likely (OR: 0.67; 95% (0.47–0.95)) to have excellent to satisfactory knowledge scores of breast cancer treatments than were those who presented with stages I & II disease (Table 8).

## Discussion

This study examined the knowledge of breast cancer risk factors, symptoms and treatment options among patients receiving breast cancer treatment at KBTH. Educational level, income level, age and cancer stage at the time of presentation were identified as predictors of patient knowledge of breast cancer.

The mean participant age for this study and late-stage diagnosis was consistent with the literature on breast cancer patient age and stage at the time of presentation in Ghana [12–14].

Our study aligns with similar reports in the literature demonstrating that only a small percentage of study participants (*n* = 51, 8.7%) had not heard of breast cancer prior to their own diagnosis [8, 15]. While this study revealed a high awareness (83%) of breast self-examination, it also revealed that the practice of breast self-examination (42%) was low. This trend has been reported among both healthcare workers and nonhealthcare workers in Sub-Saharan Africa [5, 16, 17]. In the absence of a national breast cancer screening programme, LMICs must encourage breast self-examination as a tool for early breast cancer detection.

While it is common among Ghanaian communities that breast cancer is not curable, the majority of the patients in this study (79%) believe that breast cancer can be cured [8]. Interestingly, 14% of the participants who were receiving treatment for breast cancer did not believe that their cancer could be cured. One would have expected that the information gathered during treatment would have changed this misconception. Perhaps this entrenched view is fueled by the poor survival outcomes of the large numbers of patients with advanced disease.

Although awareness about the existence of breast cancer was high among our study participants, there was a deficit in knowledge about breast cancer risk factors and symptoms. This could perhaps be expected given the late stage at the time of diagnosis of the majority of the patients. These findings from our patient cohort are similar to those of another community in Ghana, where 56% of participants were unable to list a single risk factor for breast cancer and only 12% knew a strong family history of the disease to be an associated risk factor [8]. Furthermore, 37% and 32% of participants in this study erroneously believed that using deodorants and placing money in one’s brassier were risk factors for developing breast cancer. Only 2% of women in this study believed breast cancer to be a spiritual disease, although a systematic review described this deeply rooted sociocultural misconception to be found in several Ghanaian communities [18].

Next, while most patients identified a breast “lump” as a concerning symptom of breast cancer, few identified additional concerning symptoms, including nipple discharge, skin changes and changes in breast size. Indeed, other studies from LMICs have shown that a breast mass is the most commonly identified symptom of breast cancer [6, 15, 19]. Ultimately, increased education on breast cancer risk factors and symptoms is an important public health priority so that women who have risk factors for breast cancer can be followed more closely and that individuals who have concerning symptoms of breast cancer can seek care expeditiously.

This study also revealed poor knowledge among breast cancer patients regarding breast cancer treatment options. This was unexpected in patients who had been diagnosed with and were receiving treatment for breast cancer. One would anticipate a better understanding of treatment options among patients, as they have received information on treatment options. Specifically, awareness of breast-conserving surgery as a treatment option was extremely low (7%). Given that fear of mastectomy is cited as a major driver of late presentation for the treatment of breast cancer in Ghana [3], increased awareness of the availability of breast-conserving surgery could encourage patients to present earlier. Improved patient education on treatment options should be addressed by health professionals, as this could improve treatment adherence.

Poor knowledge of risk factors and symptoms of breast cancer were shown to be associated with age older than 60 years (Tables 3, 4, 7 and 8) in our study as well as in the medical literature [20]. Considering that breast cancer risk increases with age, it is imperative that older women in Ghana be included in breast cancer awareness campaigns. This study additionally revealed that education level was associated with knowledge of breast cancer risk factors (Table 3), breast cancer symptoms (Table 4) and breast cancer treatment options (Table 5). Specifically, tertiary-level education was a predictor of satisfactory-to-excellent knowledge of breast cancer risk factors (Table 6), symptoms of breast cancer (Table 7) and treatment options (Table 8). Higher education levels have been linked to better knowledge of all aspects of breast cancer [6, 7, 21]. Similarly, higher income levels were associated with better knowledge in all domains of breast cancer in our study (Tables 3, 4, 5, 6, 7 and 8). A higher socioeconomic status is expected to increase people’s knowledge and health consciousness. Finally, knowledge of symptoms of breast cancer was inferior in patients who presented with late-stage disease (stages III and IV) (Tables 4 and 7), and treatment options (Tables 5 and 8) imply that advanced disease is a predictor of poor knowledge of the signs, symptoms and treatment options of the disease. This poor knowledge is most likely what leads to late presentation and hence advanced cancer stage. A better understanding of breast cancer among those with stage I and II disease may be responsible for their earlier presentation.

During the month of breast cancer awareness in October, educational messages are carried across both traditional and social media outlets. This study revealed that the most popular sources of information on breast cancer among study participants were TV (63.4%) and radio (44.6%), as reported in other LMICs [19]. Very few participants in this study cited print media or the internet as a resource for their knowledge about breast cancer. However, it is again worth noting the mean age of our study’s participants (50 years) and acknowledging that different generations are likely to utilise different sources of information. For example, a previous Ghanaian study found social media, teachers and electronic media to be the most commonly cited sources of information regarding breast cancer among university students [15]. Additionally, the fact that most participants in this study had less than a tertiary education and reported a low monthly income may also explain why print media and the internet were less patronised by the patients in this study.

The effectiveness of mass media in health education and health promotion has been established, with TV being the most effective and popular medium in LMICs [22]. Radio also has a large audience [8, 19]. Efforts in awareness campaigns through mass media should be intensified in Ghana via TV and radio outlets to reach more people, especially those most at risk for developing breast cancer. Approximately, a quarter of the participants also gleaned information from family and friends. This finding is also observed in other studies [6, 8, 15] and is an indication that, in this setting, information and opinions about the community are important and may influence health-seeking behaviour.

Interestingly, most of the findings of this study are not dissimilar to findings among non breast cancer patients in Ghana. This highlights the need for education among all groups of women. Importantly, improved education of breast cancer patients could, specifically, improve treatment adherence. The information needs of breast cancer patients have been identified, and education about recommended treatments and treatment options is recommended [23]. Additionally, a strategy of recent awareness campaigns in Ghana has been to involve breast cancer survivors at the forefront to share their first-hand experience with the disease, its treatment and survival, with the hope of improving breast cancer awareness among the general public. Anecdotally, the recent involvement of breast cancer survivors as a support for newly diagnosed breast cancer patients has been effective in encouraging compliance with treatment at KBTH. Breast cancer patients and survivors are potential resources that can be educated and equipped to play a unique role in awareness creation. When armed with accurate information that can be disseminated to family and friends, these patients become important breast cancer advocates in the community.

## Conclusion

This study revealed poor knowledge about breast cancer risk factors, breast cancer symptoms and breast cancer treatment options among breast cancer patients receiving treatment at a tertiary facility for breast cancer care in Ghana. This study identified higher education level and higher monthly income as predictors of better knowledge of all aspects of breast cancer, while age >60 years and advanced disease stage are predictors of poor knowledge of breast cancer.

Breast cancer awareness campaigns in Ghana need to be intensified and tailored to reach all social groups, particularly those of lower socioeconomic status and elderly individuals. Future studies are recommended in these groups to explore the factors contributing to the low levels of knowledge and their specific knowledge gaps. This will help in addressing their peculiar needs. Accurate information on TV and the radio will have the widest coverage for improving the knowledge available about breast cancer. Educational messages should emphasize symptoms of breast cancer and the availability of breast-conserving surgery as a treatment option for early disease, and they should also dispel misconceptions about the causes of breast cancer. Healthcare services should include deliberate efforts to educate breast cancer patients on the disease and treatment to improve patients’ understanding of their diagnosis, increase treatment adherence and enable patients to become breast cancer advocates within their communities.

Limitations of this study include the use of a cross-sectional design which cannot explore changes in knowledge that are likely to evolve over the treatment period. Recall bias may also have been introduced as participants were already diagnosed with breast cancer and may be further biased by the use of an interviewer-administered questionnaire. However, training on the administration of questionnaires was designed to minimize interviewer bias. Findings from this single centre may not be generalizable for all breast cancer patients in the country and is not a reflection of people who do not have a cancer diagnosis.

## List of abbreviations

GHS, Ghana Cedi; JHS, Junior High School; KBTH, Korle Bu Teaching Hospital; LMIC, Low- and middle-income countries; OPD, Our Patient Department; SHS, Senior High School; TV, Television; USD, United States Dollar.

## Conflicts of interest

Kirstyn E Brownson: consultant for ImpediMed Surgeon Advisory Board for Lymph Edema Management.

All the other authors have no conflicts of interest to declare.

## Institutional review

The study protocol was reviewed and approved by the Korle Bu Teaching Hospital Institutional Review Board (KBTH-STC/IRB/00099/2021). Written informed consent was obtained from all participants, and the research was conducted in accordance with standard research ethical principles. To ensure confidentiality, participants’ identifying details were not used.

## Figures and Tables

**Table 1.** Knowledge of breast cancer (risk factors, symptoms and treatment options).

			Total
Variable	Frequency	Percentage	*n* = 636
Knowledge of risk factors			
Family history			
No	384	60.4	
Yes	205	32.2	
No response	47	7.4	
Personal history			
No	579	91.0	
Yes	10	1.6	
No response	47	7.4	
Hormone replacement therapy			
No	568	89.3	
Yes	21	3.3	
No response	47	7.4	
Not breast feeding			
No	555	87.3	
Yes	34	5.3	
No response	47	7.4	
Not having children			
No	563	88.5	
Yes	26	4.1	
No response	47	7.4	
Early menarche			
No	578	90.9	
Yes	11	1.7	
No response	47	7.4	
Late menopause			
No	579	91.0	
Yes	10	1.6	
No response	47	7.4	
Spiritual			
No	578	90.9	
Yes	11	1.7	
No response	47	7.4	
Money in brassier			
No	386	60.7	
Yes	203	31.9	
No response	47	7.4	
Deodorant			
No	170	26.7	
Yes	235	36.9	
No response	231	36.3	
Knowledge of symptoms			
Breast lump			
No	5	0.8	
Yes	482	75.8	
No response	149	23.4	
Skin changes			
No	412	64.8	
Yes	75	11.8	
No response	149	23.4	
Nipple discharge			
No	404	63.5	
Yes	83	13.1	
No response	149	23.4	
Change in breast size			
No	450	70.8	
Yes	37	5.8	
No response	149	23.4	
Knowledge of treatment options			
Mastectomy			
No	187	29.4	
Yes	314	49.4	
No response	135	21.2	
Breast conservation surgery			
No	458	72.0	
Yes	43	6.8	
No response	135	21.2	
Endocrine therapy			
No	428	67.3	
Yes	73	11.5	
No response	135	21.2	
Chemotherapy			
No	96	15.1	
Yes	405	63.7	
No response	135	21.2	
Radiation			
No	222	34.9	
Yes	279	43.9	
No response	135	21.2	

**Table 2.** Sociodemographic characteristics of the study participants.

Demo	Frequency	Percentage
	*n*	%
Age		
≤39	97	15.3
40–49	171	26.9
50–59	187	29.4
60+	181	28.4
Highest educational level		
No formal education	53	8.3
Primary	55	8.7
Middle/JHS	162	25.5
Secondary/Senior High School (SHS)	163	25.6
Tertiary	203	31.9
Marital status		
Never married	77	12.1
Married	358	56.3
Cohabiting	7	1.1
Divorced/Separated	98	15.4
Widowed	96	15.1
Usual place of residence		
Urban	560	88.1
Peri-urban	60	9.4
Rural	16	2.5
Average monthly income (GHS)		
<500	238	37.7
500–1,000	176	27.9
1,001–2,000	85	13.5
2,001–5,000	92	14.6
>5,000	40	6.3
Cancer stage		
Stage I & II	229	38.9
Stage III & IV	360	61.1

**Table 3.** Association of sociodemographic characteristics of breast cancer patients with knowledge of breast cancer risk factors.

Variable	Knowledge of breast cancer risk factors				
	Poor	Satisfactory	Excellent	Total	*p* value
	*n* (%)	*n* (%)	*n* (%)	*n*	
Age					0.104
≤39	80 (87.9)	8 (8.8)	3 (3.3)	91	
40–49	142 (89.9)	14 (8.9)	2 (1.3)	158	
50–59	161 (93.1)	11 (6.4)	1 (0.6)	173	
60+	160 (95.8)	7 (4.2)	0 (0.0)	167	
Highest educational level					**<0.001**
No formal education	44 (95.6)	1 (2.2)	1 (2.2)	46	
Primary	51 (98.1)	1 (4.9)	0 (0.0)	52	
Middle/JHS	145 (93.5)	10 (6.5)	0 (0.0)	155	
Secondary/SHS	145 (97.3)	4 (2.7)	0 (0.0)	149	
Tertiary	158 (84.5)	24 (12.8)	5 (2.7)	187	
Marital status					0.311
Never married	66 (91.7)	6 (8.3)	0 (0.0)	72	
Married	306 (90.5)	26 (7.7)	6 (1.8)	338	
Divorced/Separated	88 (94.6)	5 (5.4)	0 (0.0)	93	
Widowed	83 (96.5)	3 (3.5)	0 (0.0)	86	
Place of residence					0.89
Urban	474 (91.8)	36 (7.0)	6 (1.2)	516	
Peri-urban	54 (94.7)	3 (5.3)	0 (0.0)	57	
Rural	15 (93.7)	1 (6.3)	0 (0.0)	16	
Average monthly income (GHS)					**0.020**
<500	210 (91.7)	13 (5.8)	1 (0.5)	224	
500–1,000	151 (94.4)	8 (5.0)	1 (0.6)	160	
1,001–2,000	68 (88.3)	9 (11.7)	0 (0.0)	77	
2,001–5,000	75 (86.2)	8 (9.2)	4 (4.6)	87	
>5,000	34 (94.4)	2 (5.6)	0 (0.0)	36	
Stage					0.854
Stage I & II	214 (93.5)	12 (5.2)	3 (1.3)	33	
Stage III & IV	338 (93.9)	19 (5.3)	3 (0.8)	360	

The bold values are statistically significant with *p* values <0.05.

**Table 4.** Association of the sociodemographic characteristics of breast cancer patients with knowledge of breast cancer symptoms.

Variable	Knowledge of symptoms of breast cancer				
	Poor knowledge	Satisfactory	Excellent	Total	*p* value
	*n* (%)	*n* (%)	*n* (%)	*n*	
Age					**<0.001**
≤39	59 (64.8)	13 (14.3)	19 (20.9)	91	
40–49	122 (77.2)	24 (15.2)	12 (7.6)	158	
50–59	145 (83.8)	14 (8.1)	14 (8.1)	173	
60+	147 (88.0)	13 (7.8)	7 (4.2)	167	
Highest educational level					**<0.001**
No formal education	45 (97.8)	0 (0.0)	1 (2.2)	46	
Primary	50 (96.1)	2 (3.9)	0 (0.0)	52	
Middle/JHS	139 (89.7)	15 (9.7)	1 (0.6)	155	
Secondary/SHS	126(84.6)	16(10.7)	7(4.7)	149	
Tertiary	113(60.4)	31(16.6)	43(23.0)	187	
Marital status					0.062
Never married	52 (72.2)	10 (13.9)	10 (13.9)	72	
Married	263 (77.8)	42 (12.4)	33 (9.8)	338	
Divorced/Separated	83 (89.3)	6 (6.4)	4 (4.3)	93	
Widowed	75 (87.2)	6 (7.0)	5 (5.8)	86	
Usual place of residence					0.166
Urban	409 (79.3)	57 (11.0)	50 (9.7)	516	
Peri-urban	48 (84.2)	7 (12.3)	2 (3.5)	57	
Rural	16 (100.0)	0 (0.0)	0 (0.0)	16	
Average monthly income (GHS)					**<0.001**
<500	209 (93.3)	14 (6.2)	1 (0.5)	224	
500–1,000	134 (83.7)	16 (10.0)	10 (6.3)	160	
1,001–2,000	57 (74.0)	7 (9.1)	13 (16.9)	77	
2,001–5,000	47 (54.0)	16 (18.4)	24 (27.6)	87	
>5,000	21 (58.3)	11 (30.6)	4 (11.1)	36	
Stage					**0.004**
Stage I & II	169 (73.8)	36 (15.7)	24 (10.5)	229	
Stage III & IV	304 (84.4)	28 (7.8)	28 (7.8)	360	

**Table 5.** Association of sociodemographic characteristics of breast cancer patients with knowledge of breast cancer treatment options.

Variable	Knowledge of breast cancer treatment options				
	Poor	Satisfactory	Excellent	Total	*p* value
	*n* (%)	*n* (%)	*n* (%)	*n*	
Age					0.479
≤39	31 (34.1)	51 (56.0)	9 (9.9)	91	
40–49	49 (31.0)	95 (60.1)	14 (8.9)	158	
50–59	71 (41.0)	89 (51.5)	13 (7.5)	173	
60+	69 (41.3)	84 (50.3)	14 (8.4)	167	
Highest educational Level					**<0.001**
No formal education	19 (41.3)	27 (58.7)	0 (0.0)	46	
Primary	29 (55.8)	21 (40.4)	2 (3.8)	52	
Middle/JHS	64 (41.3)	81 (52.3)	10 (6.4)	155	
Secondary/SHS	61 (40.9)	77 (51.7)	11 (7.4)	149	
Tertiary	47 (25.1)	113 (60.4)	27 (14.4)	187	
Marital status					0.519
Never married	24 (33.4)	41 (56.9)	7 (9.7)	72	
Married	119 (35.2)	187 (55.3)	32 (9.5)	338	
Divorced/Separated	41 (44.1)	45 (48.4)	7 (7.5)	93	
Widowed	36 (41.9)	46 (53.5)	4 (4.6)	86	
Place of residence					0.184
Urban	192 (37.2)	278 (53.9)	46 (8.9)	516	
Peri-urban	18 (31.6)	35 (61.4)	4 (7.0)	57	
Rural	10 (62.5)	6 (37.5)	0 (0.0)	16	
Average monthly income (GHS)					**0.001**
<500	99 (44.2)	112 (50.0)	13 (5.8)	224	
500–1,000	67 (41.9)	82 (51.2)	11 (6.9)	160	
1,001–2,000	23 (29.9)	48 (62.3)	6 (7.8)	77	
2,001–5,000	22 (25.3)	53 (60.9)	12 (13.8)	87	
>5,000	8 (22.2)	20 (55.6)	8 (22.2)	36	
Stage					**0.015**
Stage I & II	69 (30.1)	138 (60.3)	22 (9.6)	229	
Stage III & IV	151 (41.9)	181 (50.3)	28 (7.8)	360	

**Table 6.** Univariate and multivariate ordinal logistic regression showing socio-demographic factors and knowledge about breast cancer risk.

Variable	Knowledge about breast cancer risk factors	
	cOR (95%CI) *p*-value	aOR (95%CI) *p*-value
Age		
≤39	Ref	Ref
40–49	0.80 (0.35–1.81) 0.598	0.85 (0.34–2.09) 0.718
50–59	0.53 (0.22–1.25) 0.148	0.79 (0.30–2.07) 0.636
60+	0.31 (0.12–0.83) 0.020	0.45 (0.13–1.58) 0.211
Religion		
Christianity	Ref	Ref
Islam	0.79 (0.24–2.67) 0.711	1.10 (0.28–4.37) 0.893
Highest educational level		
No formal education	Ref	Ref
Primary	0.42 (0.04–4.82) 0.488	0.47 (0.04–5.69) 0.555
Middle /JHS	1.48 (0.31–7.00) 0.622	2.14 (0.42–11.07) 0.362
Secondary/SHS	0.59 (0.11–3.36) 0.556	0.76 (0.12–4.62) 0.762
Tertiary	3.99 (0.92–17.41) 0.065	6.27 (1.14–34.39) **0.035**
Marital status		
Never married	Ref	Ref
Married	1.17 (0.47–2.91) 0.734	1.59 (0.59-4.27) 0.362
Divorced/Separated	0.63 (0.18–2.14) 0.458	0.78 (0.21–2.88) 0.709
Widowed	0.40 (0.10–1.66) 0.207	0.94 (0.19–4.53) 0.936
Usual place of residence		
Urban	Ref	Ref
Peri-urban	0.62 (0.19–2.07) 0.440	0.80 (0.22–2.88) 0.732
Rural	0.75 (0.10–5.55) 0.778	0.51 (0.05–5.82) 0.590
Average monthly household income (GHS)		
<500	Ref	Ref
500–1,000	0.90 (0.38–2.12) 0.804	0.65 (0.25–1.70) 0.382
1,001–2,000	1.96 (0.81–4.72) 0.134	0.92 (0.31–2.71) 0.873
2,001–5,000	2.49 (1.10–5.62) **0.028**	0.98 (0.33–2.85) 0.964
>5,000	0.88 (0.19–4.04) 0.868	0.45 (0.08–2.47) 0.356
Stage		
Stage I & II	Ref	Ref
Stage III & IV	0.92 (0.47–1.82) 0.821	1.02 (0.47–2.21) 0.967

**Table 7.** Univariate and multivariate ordinal logistic regression showing socio-demographic characteristics with signs of breast cancer.

Variable	Knowledge on breast cancer signs	
	cOR (95%CI) *p*-value	aOR (95%CI) *p*-value
Age		
≤39	**Ref**	**Ref**
40–49	0.50 (0.28–0.87) 0.015	0.68 (0.36–1.28) 0.232
50–59	0.34 (0.19–0.61) 0.001	0.75 (0.37–1.49) 0.406
60+	0.24 (0.13–0.44) **0.001**	0.34 (0.16–0.72) **0.005**
Religion		
Christianity	**Ref**	**Ref**
Islam	0.37 (0.13–1.05) 0.062	0.52 (0.16–1.67) 0.271
Highest educational level		
No formal		
Education	**Ref**	**Ref**
Primary	1.75 (0.15–19.9) 0.653	1.68 (0.15–19.55) 0.676
Middle /JHS	4.93 (0.64–38.18) 0.127	3.44 (0.43–27.51) 0.244
Secondary/SHS	7.97 (1.05–60.70) 0.045	4.57 (0.58–36.04) 0.150
Tertiary	31.03 (4.18–230.1) **0.001**	10.44 (1.32–82.73) **0.026**
Marital status		
Never married	**Ref**	**Ref**
Married	0.73 (0.41–1.29) 0.280	0.84 (0.43–1.62) 0.595
Divorced/Separated	0.31 (0.13–0.71) 0.006	0.43 (0.17–1.09) 0.076
Widowed	0.38 (0.17–0.85) **0.019**	0.89 (0.34–2.33) 0.808
Usual place of residence		
Urban	**Ref**	**Ref**
Peri-urban	0.52 (0.25–1.08) 0.078	0.78 (0.34–1.76) 0.546
Average monthly income		
<500	**Ref**	**Ref**
500–1,000	2.76 (1.41–5.39) **0.003**	2.05 (1.00–4.21) 0.050
1,001–2,000	5.41 (2.61–11.23) **0.001**	2.92 (1.29–6.65) **0.010**
2,001–5,000	13.09 (6.72–25.51) **0.001**	5.25 (2.41–11.43) **0.001**
>5,000	8.87 (3.92–20.10) **0.001**	3.77 (1.49–9.54) **0.005**
Stage		
Stage I & II	**Ref**	**Ref**
Stage III & IV	0.54 (0.36–0.81) **0.003**	0.68 (0.43–1.07) 0.096

**Table 8.** Univariate and multivariate ordinal logistic regression showing socio-demographic characteristics with knowledge about breast cancer treatment.

Variable	Knowledge on breast cancer treatment options	
	cOR (95%CI) *p*-value	aOR (95%CI) *p*-value
Age		
≤39	Ref	Ref
40–49	1.08 (0.65–1.79) 0.759	1.52 (0.88–2.64) 0.132
50–59	0.74 (0.45–1.22) 0.235	1.17 (0.67–2.05) 0.574
60+	0.75 (0.45–1.23) 0.255	1.56 (0.62–2.15) 0.648
Religion		
Christianity	Ref	Ref
Islam	0.64 (0.35–1.15) 0.133	0.83 (0.44–1.57) 0.576
Highest educational level		
No formal education	Ref	Ref
Primary	0.64 (0.30–1.38) 0.258	0.68 (0.31–1.51) 0.349
Middle /JHS	1.15 (0.61–2.14) 0.671	1.13 (0.58–2.20) 0.713
Secondary/SHS	1.18 (0.63–2.23) 0.599	1.12 (0.57–2.20) 0.744
Tertiary	2.52 (1.35–4.71) **0.004**	1.88 (0.91–3.89) 0.089
Marital status		
Never married	Ref	Ref
Married	0.93 (0.57–1.53) 0.778	0.94 (0.55–1.60) 0.817
Divorced/Separated	0.65 (0.36–1.19) 0.162	0.67 (0.35–1.28) 0.227
Widowed	0.66 (0.36–1.22) 0.184	0.83 (0.41–1.62) 0.561
Usual place of residence		
Urban	Ref	Ref
Peri-urban	1.16 (0.68–1.97) 0.586	1.41 (0.81–2.46) 0.225
Rural	0.33 (0.12–0.92) 0.034	0.36 (0.12–1.05) 0.061
Average monthly income (GHS)		
<500	Ref	Ref
500–1,000	1.11 (0.75–1.65) 0.600	0.94 (0.61–1.43) 0.763
1,001–2,000	1.73 (1.04–2.87) **0.034**	1.28 (0.72–2.27) 0.403
2,001–5,000	2.40 (1.46–3.95) **0.001**	1.44 (0.79–2.60) 0.231
>5,000	3.52 (1.69–7.31) **0.001**	2.09 (0.94–4.66) 0.072
Stage		
Stage I & II	Ref	Ref
Stage III & IV	0.63 (0.46–0.88) **0.006**	0.67 (0.47–0.95) **0.023**

## References

[1] Cancer Today. https://gco.iarc.who.int/today/.

[2] Sedeta ET, Jobre B, Avezbakiyev B (2023). Breast cancer: global patterns of incidence, mortality, and trends. JCO.

[3] Clegg-Lamptey J, Dakubo J, Attobra YN (2009). Why do breast cancer patients report late or abscond during treatment in ghana? A pilot study. Ghana Med J.

[4] Khan MA, Hanif S, Iqbal S (2015). Presentation delay in breast cancer patients and its association with sociodemographic factors in North Pakistan. Chin J Cancer Res.

[5] Udoh RH, Tahiru M, Ansu-Mensah M (2020). Women’s knowledge, attitude, and practice of breast self- examination in sub-Saharan Africa: a scoping review. Arch Public Health.

[6] Prusty RK, Begum S, Patil A (2020). Knowledge of symptoms and risk factors for breast cancer among women: a community based study in a low socioeconomic area of Mumbai, India. BMC Women’s Health.

[7] Opoku SY, Benwell M, Yarney J (2012). Knowledge, attitudes, beliefs, behavior and breast cancer screening practices in Ghana, West Africa. Pan Afr Med J.

[8] Dadzi R, Adam A (2019). Assessment of knowledge and practice of breast self-examination among reproductive age women in Akatsi South district of Volta region of Ghana. PLoS One.

[9] Agbokey F, Kudzawu E, Dzodzomenyo M (2019). Knowledge and health seeking behavior of breast cancer patients in Ghana. Int J Breast Cancer.

[10] Asoogo C, Duma SE (2015). Factors contributing to late breast cancer presentation for health care among women in Kumasi, Ghana. Curationis.

[11] Calys-Tagoe BN, Brownson K, Nsaful J (2024). Satisfaction levels and associated influencing factors among inpatients and outpatients with breast cancer at a tertiary health facility in Ghana. Ghana Med J.

[12] Mensah AC, Yarney J, Nokoe SK (2016). Survival outcomes of breast cancer in Ghana: an analysis of clinicopathological features. OALib.

[13] Ayettey H, Yarney J, Sanuade O (2021). Neoadjuvant or adjuvant chemotherapy for breast cancer in Sub-Saharan Africa: a retrospective analysis of recurrence and survival in women treated for breast cancer at the Korle Bu Teaching Hospital in Ghana. JCO Glob Oncol.

[14] Ssentongo P, Oh JS, Amponsah-Manu F (2022). Breast cancer survival in Eastern Region of Ghana. Front Public Health.

[15] Osei-Afriyie S, Addae AK, Oppong S (2021). Breast cancer awareness, risk factors and screening practices among future health professionals in Ghana: a cross-sectional study. PLoS One.

[16] Hamed E, Alemrayat B, Syed MA (2022). Breast cancer knowledge, attitudes and practices among women in Qatar. Int J Environ Res Public Health.

[17] Altirifi HI, Elsanousi OM, Bedri S (2022). Very poor practices regarding breast cancer screening among Sudanese female workers at a secondary-level hospital: a cross-sectional study. Pan Afr Med J.

[18] Afaya A, Anaba EA, Bam V (2024). Sociocultural beliefs and perceptions influencing diagnosis and treatment of breast cancer among women in Ghana: a systematic review. BMC Women’s Health.

[19] Younis M, Al-Rubaye D, Haddad H (2016). Knowledge and awareness of breast cancer among young women in the United Arab Emirates. Adv Breast Cancer Res.

[20] Grunfeld EA, Ramirez AJ, Hunter MS (2002). Women’s knowledge and beliefs regarding breast cancer. Br J Cancer.

[21] Wardle J, Waller J, Brunswick N (2001). Awareness of risk factors for cancer among British adults. Public Health.

[22] Gupta YK, Sharma SK Mass Media for Health Education (A Study in the State of Rajasthan.

[23] Lu H, Xie J, Gerido LH (2020). Information needs of breast cancer patients: theory-generating meta-synthesis. J Med Internet Res.

